# Large Cutting Depth and Layered Milling of Titanium Alloy Thin-Walled Parts

**DOI:** 10.3390/ma13071499

**Published:** 2020-03-25

**Authors:** Jun Zha, Jianxin Liang, Yipeng Li, Huijie Zhang, Yaolong Chen

**Affiliations:** 1 School of Mechanical Engineering, Xi’an Jiaotong University, Xi’an 710049, China; liangjianxin@stu.xjtu.edu.cn (J.L.); liyipeng@mail.xjtu.edu.cn (Y.L.); zhanghuijie@xjtu.edu.cn (H.Z.); chenzwei@mail.xjtu.edu.cn (Y.C.); 2 Shenzhen Research School, Xi’an Jiaotong University, Shenzhen 518057, China

**Keywords:** thin-walled parts, large-cutting depth, layered milling, titanium alloy

## Abstract

Deformation of thin-walled titanium alloys can occur during the milling process due to the cutting force and chatter vibration, which can influence the precision of the finished parts. In this research, a new milling method without auxiliary support for machining of thin-walled parts was proposed. A large cutting depth and layered milling technology were used during rough machining, with a different machining allowance for each subsequent remaining layer. In the finishing stage, the surface of the previous layer needed to be dressed before processing the next layer. A TiAlSiN-coated, cemented carbide milling cutter was used to machine titanium alloy thin-walled parts, which are characterized by continuous multilayers of unequal thickness. The processing path was simulated using HyperMILL software, and the machining accuracy was detected by the 3D optical scanner. The measurement results indicated that the surface contour accuracy of the parts was ±0.21 mm, within a range of ±0.30 mm. The machining efficiency was increased by 40%, while guaranteeing machining accuracy.

## 1. Introduction

Titanium alloys have various applications in the aerospace field due to their high strength, high-temperature resistance, corrosion resistance, and low-temperature brittleness [1]. The proportion of titanium alloy in thin-walled parts has been increasing with the increasing demand for performance in the aerospace manufacturing field. Titanium alloy thin-walled parts have low structural rigidity and a large amount of metal removal. It is easy to produce machining deformation, leading to difficulty in controlling machining accuracy due to the influence of cutting force, cutting chatter, and other factors during the process of machining thin-walled parts. At the same time, titanium alloys are recognized as difficult-to-machine materials because of their low modulus of elasticity, poor thermal conductivity, and high chemical activity. Titanium alloys have low machining efficiency and poor surface quality after machining and are prone to causing sticking of cutting tools during processing.

A large number of scholars have focused on the problems of quality and efficiency in the machining of thin-walled parts. Some researchers have carried out research to improve the machining accuracy by adding auxiliary support and improving the machining rigidity of the parts. By analyzing micromilling mechanics, Zhaojun Kou et al. found that the axial force produced on a U-shaped thin wall structure had a significant impact on the deformation of thin-walled parts. Therefore, the method of low melting point alloy auxiliary support was used to balance the axial force in the cutting process to improve the cutting rigidity of the thin-walled parts. The feasibility of this method was verified by machining a 15-mm-thick beryllium bronze workpiece [2].

Reasonable planning of the tool machining path and use of the proper machining tool have important impacts on the machining accuracy of parts. Hence, Chen et al. proposed an active compensation method during the process of machining by using an iterative calculation method. The relationship between the cutting force and deformation was considered to compensate for the machining errors of each layer. Through the simulation experiment, it was concluded that the machining errors were reduced when using the active compensation method [3]. Rai et al. established a comprehensive verification model for the high-speed milling of thin-walled parts based on finite element methods. The prediction results of elastic-plastic deformation and machining quality by carrying out cutting simulation on the process parameters, processing paths, tool selection, and other factors in the process of machining were used to guide the actual machining. The accuracy of the model was verified by the actual machining [4]. Liu et al. determined that machining accuracy will be affected by the milling thickness during the processing. A finite element model of cutting force based on the position change of the positioning element was proposed by studying the optimization of the positioning element on the secondary positioning surface. The position of the positioning device during the milling process of the thin-walled parts was, thus, optimized. The maximum deformation of the thin-walled parts could be reduced from 0.064 to 0.047 mm by using this method [5]. Herranz et al. optimized the cutting conditions and chip load by simulating methods at different machining stages to reduce the instability during machining and improve the machining accuracy [6]. Calleja et al. minimized the error between the design surface and the conical envelope to reduce machining errors when using ball-end milling cutters for five-axis machining [7]. Li et al. proposed a new trochoidal milling method that can effectively improve machining efficiency [8]. Bo et al. proposed a new five-axis side computer numerical control (CNC) machining method, with which the best machining path was sought through global optimization of the tool shape and its movement [9].

The modeling method can effectively simulate cutting stability during the cutting process and provide theoretical support for the optimization of cutting parameters. Urbikain et al. predicted cutting flutter, cutting force, and surface morphology during the cutting process by modeling the cutting process, providing theoretical guidance for optimization and selection of cutting parameters and selection of cutting tools. The stability map of the steering system at a very low speed (<100 rpm) was obtained using MATLAB to guide the machining and to improve the stability of the turning system. Based on the cutting conditions, a time-domain surface profile prediction model for a side milling cutter with a circular end mill was established, and the accuracy of the model was verified. A linear stability model for calculating the dynamic displacement coefficient according to the tool vibration frequency was established, and the results of the model were supported by different data. A lathe flutter model was established by the SFM method and the modern configuration method of the Chebyshev polynomial, which was used to select the threshold of cutting parameters and improve the machining efficiency. Based on the dynamic cutting force, a contour map of stability was drawn and a time-domain model was obtained to predict milling stability [10,11,12,13,14]. Campa et al. predicted the stability problems during machining by establishing a three-dimensional dynamic model of the stability convex corners of low-rigidity parts, and provided guidance for parameter optimization and tool selection. An average method was used to establish the stability model of a flexible milling system. Based on this, a calculation method for the stability chart was proposed to select the order of the tool spindle speed during the machining process [15,16]. Arizmendi et al. established a model for predicting the surface topography of milling based on the tool vibration problem during cutting [17]. Bravo et al. proposed a method for obtaining instability or stability convex corners, which is used for mechanical structures and processed workpieces with similar dynamic behavior. The accuracy of the method was verified by actual machining [18]. Zhang et al. established a workpiece deformation error model based on the feedback mechanism between milling force and milling error. Based on the R-F (Reckwitz- Fiessler) and Edge worth reliability analysis methods, a machining accuracy reliability model was established [19].

In addition, appropriate process parameters can improve the surface integrity of a part and improve the machining accuracy. Therefore, Thepsonthi et al. established a model of machining process parameters and surface quality based on the statistical multi-objective particle swarm optimization method to determine the optimal cutting process parameters during the milling process. The cutting depth was the most important factor affecting the top burr, and the feed rate and feed rate per tooth had negative effects on surface roughness [20]. Rituparna Datta et al. proposed a multi-objective genetic algorithm based on cutting process parameters and established a mathematical model of the relationships between the cutting process parameters and generation time, production cost, and surface roughness. The non-dominated sorting genetic algorithm (NSGA-II) was used to solve the optimal solution of the processing problem, and the ε-constrained single-objective genetic algorithm and classical optimization method (SQP) were used to test the optimal solution [21]. Soroush Masoudi et al. studied the influence of residual stress on machining quality during cutting processing. By studying the relationships between cutting force, cutting temperature, residual stress, and deformation, they indicated that the cutting force and temperature had direct influence on the residual stress and deformation of thin-walled parts [22]. 

Special processing methods can also effectively improve the surface quality of thin-walled parts. Alexander et al. studied the application of electron beam melting (EBM) technology in the manufacture of thin-walled parts. They reduced the anisotropy by changing the microstructure of the parts and used additives to improve machining accuracy. In order to ensure high processing quality, it is better to use curved surfaces than flat surfaces in the design stage of thin-walled parts [23]. A substantial number of studies have examined the processing of thin-walled parts, but most have focused on the finishing stage. To date, no overall solution has been proposed for the entire process of part production, from the beginning of the blank to the final forming stage.

In order to ensure the machining efficiency and accuracy of thin-walled parts in the process of machining, it is necessary to conduct in-depth research from the perspectives of the cutting process parameters, tool paths, and machining processes, among others [24]. The static normal force is the most sensitive to tool wear in up-milling and down-milling, which has a negative effect on the contour accuracy of milled hardened steel [25]. In this research, we propose a new processing technology based on a typical thin-walled titanium alloy part. A large cutting depth and layered milling technology were used in the rough machining, with a different machining allowance for each subsequent remaining layer. In the finishing stage, a one-shot forming method without auxiliary support was used. The surface of the previous layer needed to be dressed before processing the next layer. The feasibility of this processing technology was then verified by actual processing. The machining efficiency was increased by 40% using this method, while also ensuring the processing quality.

## 2. Materials and Methods

### 2.1. Experimental Workpiece

**Figure 1:**
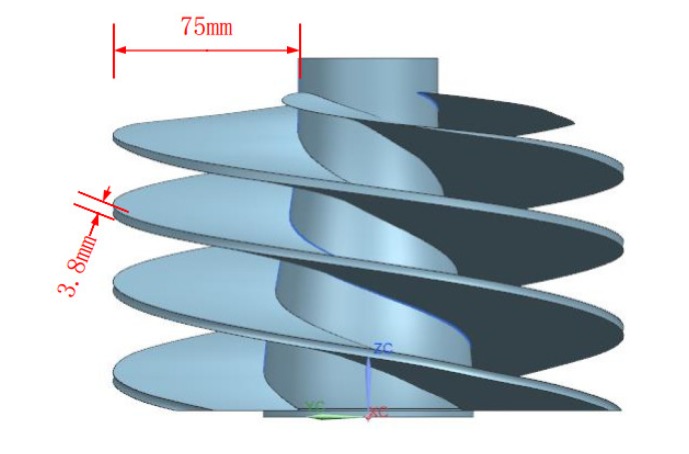
Workpiece model.

In this research, typical titanium alloy thin-walled parts were selected for machining, as shown in Figure 1. The size of the workpiece blank was φ 210 mm × 200 mm, and the accuracy requirements for the outline dimensions were ±0.30 mm. The workpiece was composed of three complex helix surfaces, the blade overhang depth was 75 mm, the wall thickness at the thinnest part was 3.8 mm, and the ratio of the depth to thickness exceeded 19 (as expected for a typical thin-walled part) [24]. In the machining process, the metal removal rate was as high as 80%. The chemical composition of the titanium alloy is shown in Table 1 and the physical and mechanical properties are shown in Table 2.

### 2.2. Experimental Conditions

Due to the complex structure of the machined parts and the high accuracy requirements, a DMG HSC-75 linear five-axis CNC center (DMG MORI, Shanghai, China) with a maximum speed of 18,000 rpm was used for the experiments. The maximum strokes of the X, Y, and Z axes were 855 mm, 600 mm, and 600 mm, respectively. The positioning accuracy and the repeated positioning accuracy of the X, Y, and Z axes were 0.005 mm and 0.003 mm, respectively. The B and C axis rotation accuracy and repeated rotation accuracy were 8” and 5”, respectively. The spindle torque was 80 N∙m and the spindle power was 35 kW. A GOM ATOS 5 industrial 3D optical scanner (GOM: ATOS Core, Braunschweig, Germany) was used to detect the machining accuracy of the workpiece. This device scanned every 0.2 s at 100 frames per second, with 12 million measurement points taken each time, with a scan accuracy of 0.028 mm. TiAlSiN-coated carbide milling cutters were used for the experiments.

### 2.3. Experimental Design

**Figure 2:**
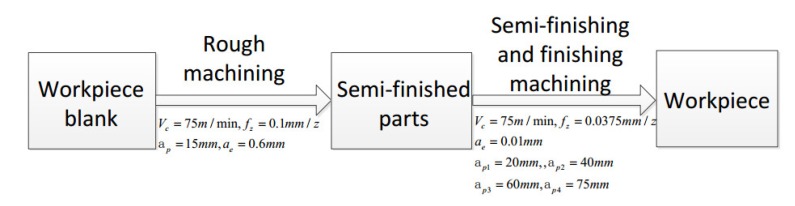
The process flow chart.

In order to ensure the machining efficiency and precision of the workpiece, the cutting process parameters and processing technology should be as reasonable as possible. A five-axis CNC milling and one-time forming process scheme without auxiliary support were proposed to reduce the positioning errors caused by multiple clamping during the cutting process in this study. The process flow of this experiment entailed roughing, semi-finishing, and finishing. According to the process planning, the appropriate cutting parameters were selected to ensure that the desired workpiece shape was obtained after each processing step. The process flow chart is shown in Figure 2.

#### 2.3.1. Rough Machining

The primary purpose of the rough machining stage is for a large amount of blank material to be removed with high efficiency. A parts contour suitable for processing was obtained to facilitate subsequent forming of part. A large cutting depth and use of high-speed dynamic milling can evenly distribute the cutting force on the cutting edge to stabilize the cutting force, while at the same time the chips can be efficiently discharged during the machining process, so that the impact of cutting heat on the tool can be reduced and the service life of the tool can be improved. The combination of a large cutting depth and other cutting process parameters meant the metal removal rate per unit time was very high, thereby guaranteeing processing efficiency [25]. A TiAlSiN-coated, cemented carbide, four-edge custom milling cutter with a diameter of 12 mm, total length of 78 mm, cutting edge length of 30 mm, and coating thickness of 1.5 μm was used for these experiments. Unequal grooving treatment was carried out at 0°, 83°, 180°, and 263° to reduce cutting flutter.

**Figure 3:**
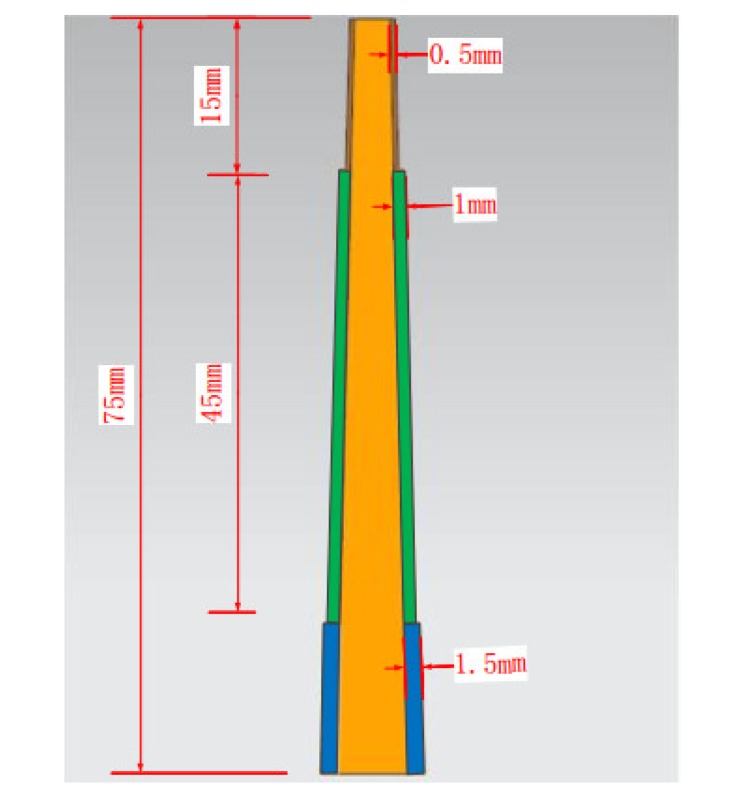
Rough machining.

In the rough machining stage, a cutting depth of 15 mm was selected based on the overhang depth of the workpiece, which was 75 mm, and the workpiece was processed in 5 steps. After processing, three stepped profiles were formed, as shown in Figure 3: with a unilateral allowance of 0.5 mm and depth of 15 mm; a unilateral allowance of 1 mm and depth of 45 mm; and a unilateral allowance of 1.5 mm and depth of 15 mm. In this figure, the yellow part is the ideal interface shape of the blade and the other parts are the machining allowance left after processing was completed, which was convenient for semi-finishing and smooth finishing.

#### 2.3.2. Semi-Finishing Machining and Finishing Machining

In order to improve the machining efficiency and ensure machining quality, a large cutting depth and layered milling method were adopted in the semi-finishing and finishing stages. The surface shape of the previous layer was trimmed when the next layer was processed, so the semi-finishing and finishing could be carried out at the same time, thus ensuring machining efficiency and accuracy.

**Figure 4:**
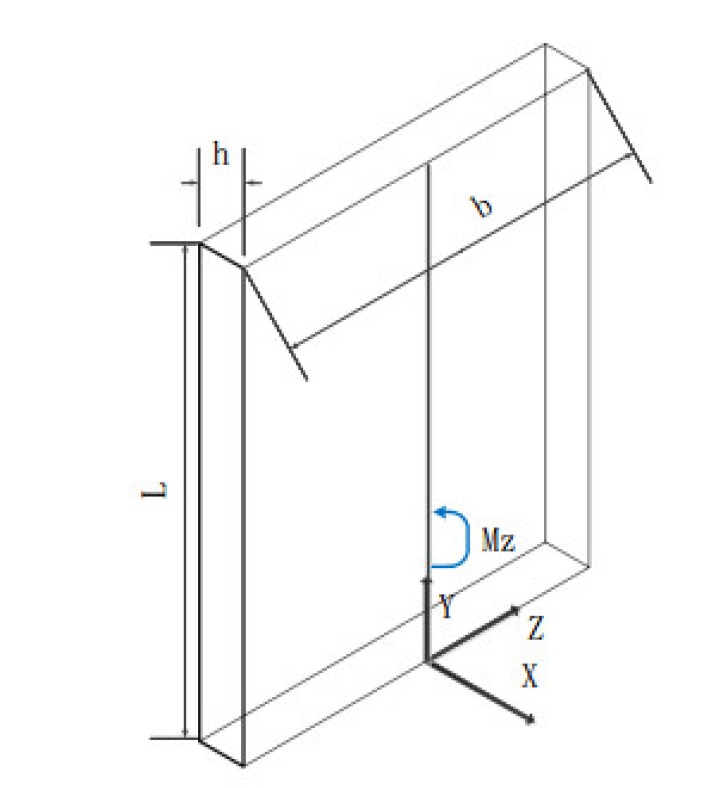
Bending moment diagram.

During the process, which featured a large cutting depth and layered milling, the cutting force on the workpiece was evenly distributed along the cutter teeth. When unfolded, the spiral blade of the workpiece was approximately equivalent to a flat plate. We can see that a uniform load *q* along the direction of the cutting edge of the tool acted on the workpiece blade. This load is approximately equivalent to a force of *qL* acting at the midpoint of the overhanging depth direction of the workpiece. The external force was simplified as a bending moment $M_{z}$ acting on the plane of the main shaft, as shown in Figure 4.

(1) $$M_{z}=\frac{qL^{2}}{2}.$$

According to the general formula of normal stress,

(2) $$\sigma_{x}=\frac{F_{N}}{A}+\frac{M_{z}y}{I_{z}}+\frac{M_{y}z}{I_{y}}$$

The normal stress at any point of the cross-section under the action of bending moment $M_{z}$ was

(3) $$\sigma_{x}=\frac{M_{z}y}{I_{y}}$$

The maximum compressive stress and the maximum tensile stress on the plate were equal when the plates had a pair of symmetrical axes perpendicular to each other:

(4) $$\sigma_{\max}=\pm \frac{M_{z}y_{\max}}{I_{z}}=\pm \frac{M_{z}}{W_{Z}}$$

(5) $$W_{z}=\frac{I_{z}}{y_{\max}}$$

For a rectangular cross-section of width h and length b, we use

(6) $$W_{z}=\frac{bh^{2}}{6}$$

From Equations (1), (3), (4), and (5), we can obtain

(7) $$\sigma_{\max}=\pm \frac{3qL^{2}}{bh^{2}}$$

The cutting force was concentrated on the outer edge of the blade when the traditional milling method was used to finish machining. It can, thus, be concluded that

(8) $$\sigma_{\max}=\pm \frac{6qL^{2}}{bh^{2}}.$$

It can be seen that the cutting force is evenly distributed on the cutting edge of the tool when using the large cutting depth side milling method, and the rigidity of the material was better than that from traditional machining, which can effectively control cutting deformation. Hence, we can use the large cutting depth and layer milling finishing method to control the deformation of thin-walled parts during processing.

According to the structural characteristics of the workpiece, the piece was divided into four layers for machining during the semi-finishing and finishing stages. The cutting process parameters were:

1. $V_{c}=75\;m/\min,f_{z}=0.0375\;mm/z,a_{p}=20\;mm,a_{e}=0.01\;mm$
2. $V_{c}=75\;m/\min,f_{z}=0.0375\;mm/z,a_{p}=40\;mm,a_{e}=0.01\;mm$
3. $V_{c}=75\;m/\min,f_{z}=0.0375\;mm/z,a_{p}=60\;mm,a_{e}=0.01\;mm$
4. $V_{c}=75\;m/\min,f_{z}=0.0375\;mm/z,a_{p}=75\;mm,a_{e}=0.01\;mm$

According to the empirical formula of the cutting force, as shown in Equation (6), the cutting forces corresponding to each of the different process parameters were 11.049, 22.230, 33.4616, and 41.9074 N.

(9) $$F=317{.6341v}_{c}{}^{-0.0135}a_{p}{}^{1.0086}a_{e}{}^{0.8316}f_{z}{}^{0.7590}.$$

**Figure 5:**
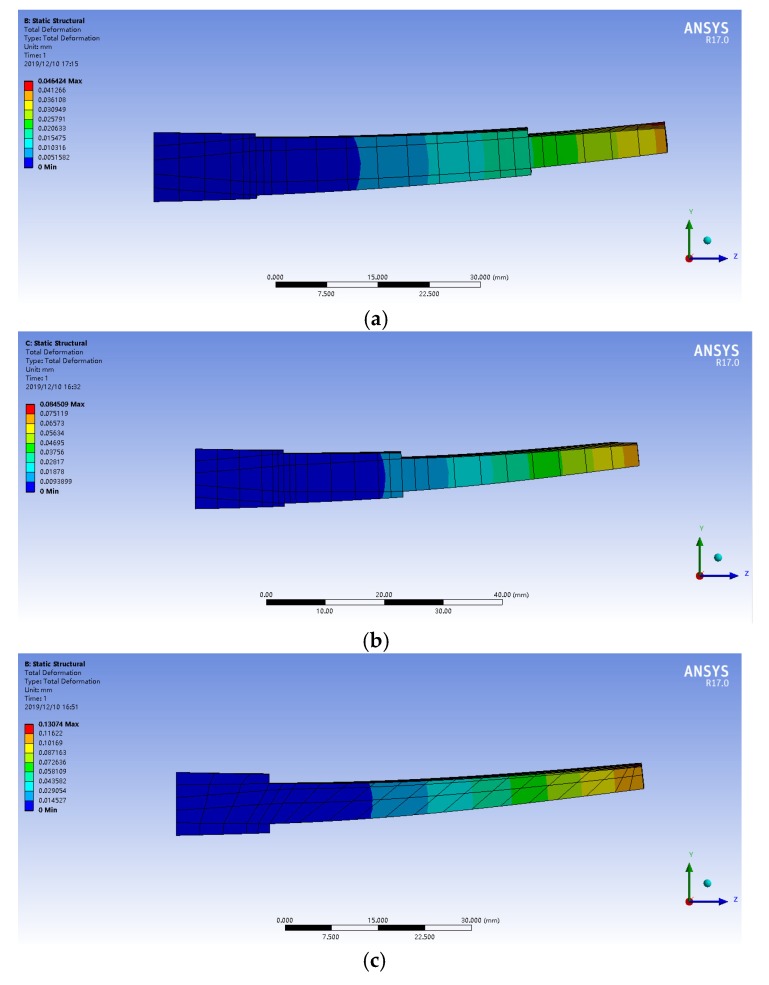

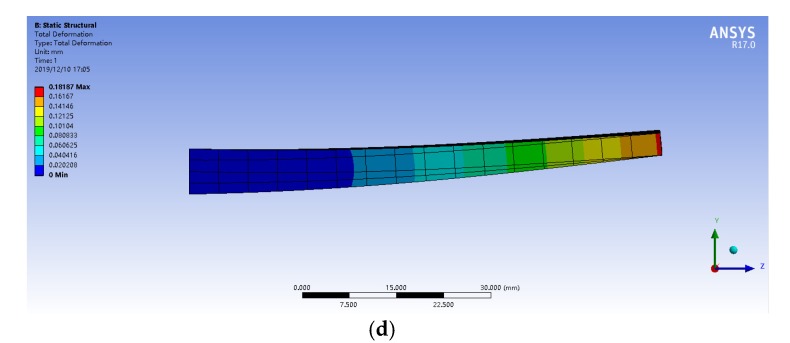
Deformation: (**a**) first layer; (**b**) second layer; (**c**) third layer; (**d**) fourth layer.

In order to verify that the deformation of the part can be effectively controlled using this machining method, the finite element simulation method was used for preliminary verification. We extended the workpiece blade along the edge of the spiral, which was approximately equivalent to a thin plate with a variable cross-section. This model was imported into ANSYS software. The material characteristics were set according to the parameters in Table 1 and Table 2 in ANSYS software, the cutting force was loaded in the middle of the workpiece along the direction of the cutting edge of the cutting tool, and a positioning constraint was set at the bottom of the workpiece to ensure that the workpiece did not move during the simulation. Deformation during machining was then simulated, as shown in Figure 5.

It can be calculated from the knowledge of material mechanics that the uniform distribution of cutting force along the cutting edge of the tool can effectively suppress the deformation of the workpiece. The simulation results show that the values for the deformation of parts were 0.046 mm, 0.084 mm, 0.130 mm, and 0.182 mm during the semi-finishing and finishing stages, which all meet the requirements for the dimensional accuracy of the contoured surfaces of the parts. Thus, this method can be used in part processing.

**Figure 6:**
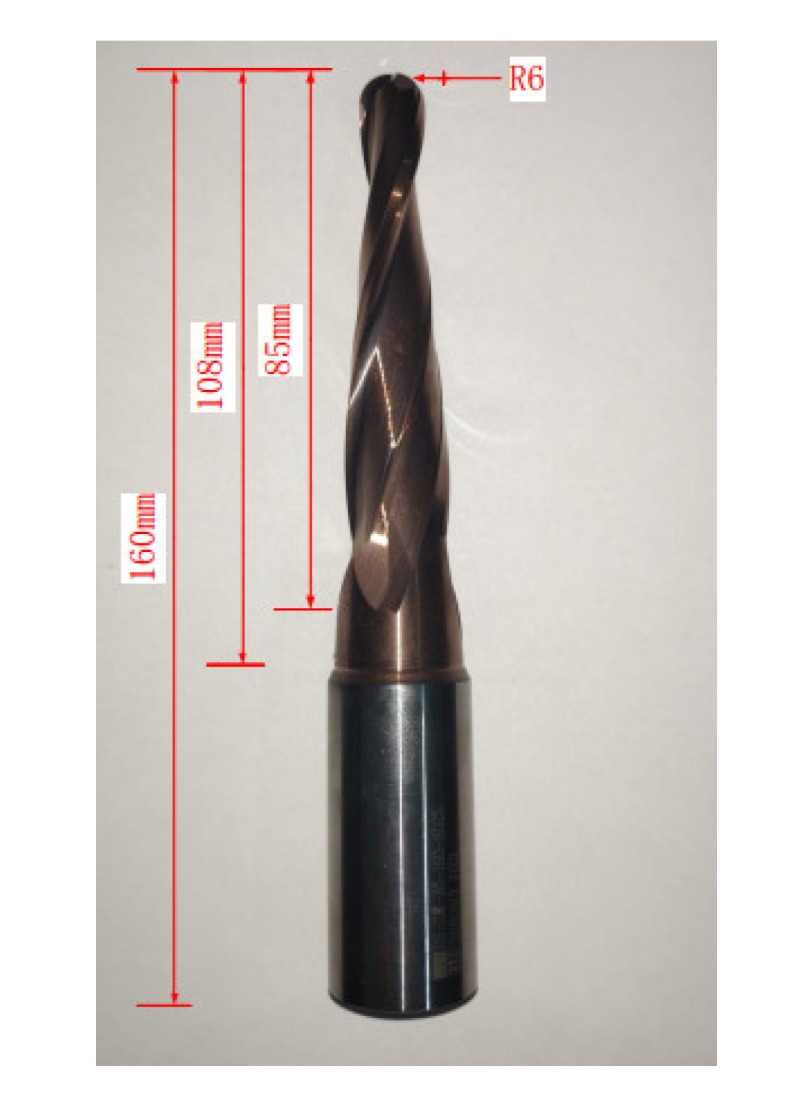
Taper milling cutter.

According to the structural characteristics of the workpiece and process planning, in order to ensure the rigidity of the tool, a TiAlSiN-coated, cemented carbide, taper milling cutter with a single side taper of 3°, a total length 160 mm, and a cutting edge length of 85 mm was used to carry out these experiments, as shown in Figure 6.

**Figure 7:**
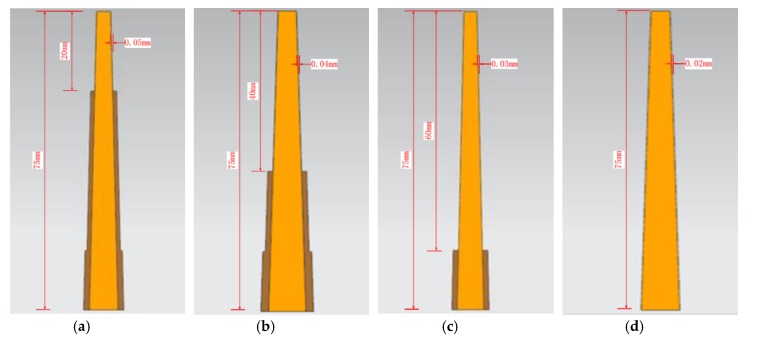
Semi-finishing and finishing machining: (**a**) first layer; (**b**) second layer; (**c**) third layer; (**d**) fourth layer.

Semi-finishing and finishing were performed in four steps. The cutting parameters were: (a) v_c_ = 75 m/min, f_z_ = 0.0375 mm/z, a_p_ = 20 mm, a_e_ = 0.01 mm; (b) v_c_ = 75 m/min, f_z_ = 0.0375 mm/z, a_p_ = 40 mm, a_e_ = 0.01 mm; (c) v_c_ = 75 m/min, f_z_ = 0.0375 mm/z, a_p_ = 60 mm, a_e_ = 0.01 mm; (d) v_c_ = 75 m/min, f_z_ = 0.0375 mm/z, a_p_ = 75 mm, a_e_ = 0.01 mm. The cutting depths (20, 40, 60, and 75 mm) and workpiece allowances (0.05 mm, 0.04 mm, 0.03 mm, and 0.02 mm) after each step are shown in Figure 7. Different processing parameters and machining allowances were selected during the process to ensure that the material had good rigidity and processing accuracy.

## 3. Machining and Measurement Results

### 3.1. Machining

**Figure 8:**
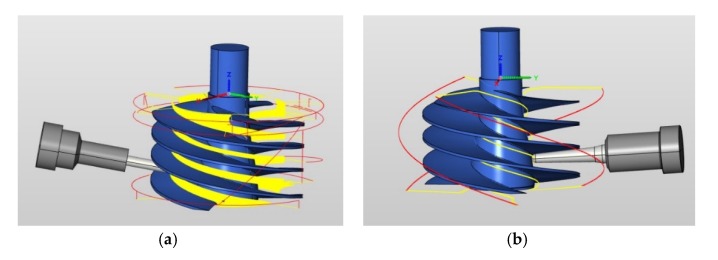
Processing path: (**a**) rough machining; (**b**) semi-finishing and finishing machining.

Emulsion cooling was used during processing and a milling method was adopted. A cycloidal milling method was used during the rough machining process and cycloid machining path was generated using HyperMILL software. The cutting parameters were: v_c_ = 75 m/min; f_z_ = 0.1 mm/z; a_p_ = 15 mm; a_e_ = 0.6 mm. A five-axis numerically controlled (NC) milling method without auxiliary support was used during semi-finishing and finishing. Cutting parameters were as described in the previous section. The processing path is shown in Figure 8.

### 3.2. Contour Accuracy Measurements

**Figure 9:**
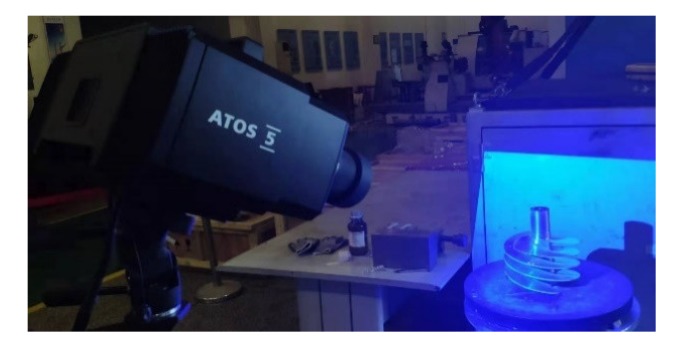
Machining accuracy measurement.

A GOM ATOS 5 industrial 3D optical scanner was used to detect the machining accuracy. In order to ensure whether the contour accuracy of the part could be accurately obtained, a layer of Ti powder was uniformly sprayed on the surface to prevent reflections. An appropriate number of calibration points was attached to the surface of the part to ensure that the measurement results could be accurately stitched together. The measurement process is shown in Figure 9.

**Figure 10:**
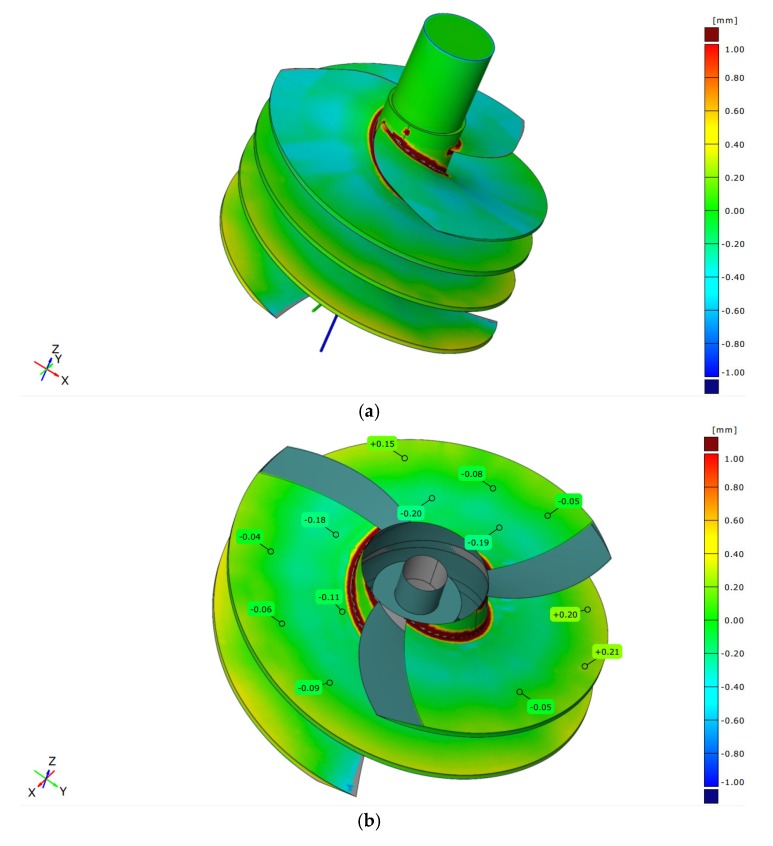
Machining accuracy measurement: (**a**) 3D model; (**b**) measurement results.

By comparing and analyzing the part model generated by 3D scanning to the original model, the surface contour accuracy of the part could be obtained, as shown in Figure 10. The part surface contour accuracy was ±0.21 mm within the range of ±0.30 mm. The deviation relative to the entire part was 0.079. The machining accuracy of the part was effectively guaranteed by selecting the milling method for thin-walled parts without auxiliary support.

## 4. Conclusions

In this paper, a new thin-walled part processing technology was proposed. The feasibility of this processing method was verified by theoretical and experimental analyses.

- In the rough machining stage, a large cutting depth and layer milling method were used to improve efficiency. It takes 87.3 h to complete rough machining under the original processing technology. However, this process took only 52.7 h when using the new processing technology; thus, the machining efficiency was increased by 40%. At the same time, the suitable surface contours of parts were provided for semi-finishing and finishing.
- In the finishing stage, a one-shot forming method was used. The surface of the previous layer needs to be dressed when processing the next layer. The surface contour accuracy of parts was within the range of ± 0.30 mm.

This technology can effectively improve the machining efficiency and accuracy of thin-walled parts by using the milling method without auxiliary support, as described in this paper. In the later stage, the corresponding mathematical model should be established to optimize the processing parameters according to different processing conditions to improve the machining efficiency further.

## Figures and Tables

**Table 1:** Chemical composition table of titanium alloys w (%).

Al	V	Fe	N	C	O	H	Ti
6.0	4.0	0.3	0.05	0.1	0.2	0.0125	Rest

**Table 2:** Physical and mechanical properties of titanium alloys.

Tensile Strength	Yield Strength	Elongation	Shrinkage
902 Mpa	824 Mpa	10%	30%

## Nomenclature

v_c_	cutting speed (m/min)
f_z_	feed per tooth (mm/z)
a_p_	cutting depth (mm)
a_e_	cutting width (mm)
F	cutting force (N)
M_z_, M_y_	bending moment (N m)
$\sigma_{x}$	normal stress (N/m^2^)
I_z_, I_y_	inertia moment (m^4^)
W_z_	modulus of flexural section (m^3^)
